# Recombinant Fusion Protein Containing Plant Nigellothionin Regulates the Growth of Food-Spoiling Fungus (*Aspergillus niger*)

**DOI:** 10.3390/foods12163002

**Published:** 2023-08-09

**Authors:** Anna S. Barashkova, Dmitry Yu. Ryazantsev, Anna S. Zhuravleva, Vladimir V. Sharoyko, Eugene A. Rogozhin

**Affiliations:** 1 Shemyakin and Ovchinnikov Institute of Bioorganic Chemistry Russian Academy of Sciences, Moscow 117937, Russia; barashkova.an@gmail.com (A.S.B.); d.yu.ryazantsev@gmail.com (D.Y.R.); 2 All-Russian Institute for Plant Protection, Pushkin 196608, Russia; 3 Agrophysical Research Institute, Saint-Petersburg 195220, Russia; zhuravlan@gmail.com; 4 Department of General and Bioorganic Chemistry, Pavlov First Saint-Petersburg State Medical University, Saint-Petersburg 197022, Russia; sharoyko@gmail.com

**Keywords:** plant antifungal peptides, recombinant fusion proteins, heterologous expression, regulatory activity, cytotoxic activity

## Abstract

This study aimed to obtain a recombinant chimeric protein named trx-NsW2 via theheterologous expression of the multifunctional antimicrobial peptide nigellothionin from black cumin (*Nigella sativa* L.) seeds in the *Escherichia coli* system. The protein was purified using a combination of Ni-NTA affinity chromatography and reversed-phase HPLC. Based on the HPLC calibration, the total yield of the protein was calculated to be 650 mg/L of bacterial culture. The fungistatic activity of trx-NsW2 against the food-spoiling fungus *Aspergillus niger* was demonstrated as itinhibited the maturation of conidiawithout affecting conidial germination or fungal growth. In contrast to mature nigellothionin NsW2, the fusion protein showeda low level of cytotoxicity towards both normal and tumor cell lines at concentrationsof up to 100–200 µM. Interestingly, at lower concentrations, it even stimulated cytokinesis. These findings are of critical importance for applying chimeric antimicrobial proteins obtained via microbiological synthesis in applied science.

## 1. Introduction

Plant antimicrobial peptides (AMPs) are crucial in various processes, including seed germination, plant growth, flowering, fruiting, etc. They are primarily defensive [1]. They primarily function as a defense mechanismsagainst biotic and abiotic stress factors such as plant–pathogen interactions, cold, drought, sunlight exposure, and soil salinity [1]. AMPs can be categorized into seven main families: thionins, defensins, non-specific lipid-transfer proteins (nsLTP), hevein-like peptides, α-hairpinins (hairpin-like peptides), snakins, and cysteine-knotted peptides (linear and cyclic) [2]. Among these families, defensins, thionins, and nsLTPsalso belong to classes of pathogenesis-related proteins (classes PR-12, -13, and -14) [2]. Additionally, there are other plant peptides that have been less studied and remain unclassified, such as proline-rich, proline-rich/hydroxyproline-rich, and glycine-rich peptides and shepheridins [2,3,4,5]. The most extensively studied function of plant AMPs is their ability to protect plants from pathogenic microorganisms, making them potential candidates for novel pesticides in agriculture and food preservatives [6]. Whilethe regulatory functions of AMPs have been described, they are not as well-studied except for defensins, which have been examined in the contexts of both plants and pathogens [7]. Plant thionins are recognized as membrane-active peptides that disrupt cell membranes and induce cell death [8]. These peptides exhibit antimicrobial activities against Gram-positive and Gram-negative bacteria, fungi, yeasts, and eukaryotic cell cultures [8,9]. They inhibit the growth of bacteria, filamentous fungi, and oomycetes, with an IC_50_ concentration ranging from 1 to 15 µg/mL [8,9]. Furthermore, thionins affect eukaryotic cell cultures (including mammals and insects) and alter the permeability of plant protoplasts [8,9,10].

Previously, we identified a group of thionins known as nigellothionins from black cumin (*Nigella sativa* L.) [11,12]. These thionins exhibit diverse biological functions, including antibacterial, antifungal, and cytotoxic activities [11,12]. In this study, we investigate the involvement of the plant thionin NsW2 (nigellothionin NsW2) from black cumin (*Nigella sativa*) in a biotechnologically engineered fusion protein called thioredoxin-NsW2 (trx-NsW2) against the food-spoiling fungus *Aspergillus niger*.

## 2. Materials and Methods

### 2.1. Microorganisms

The *Aspergillus niger* strain INA 00760, the *A. fumigatus* strain KPB F-37, and the*Candida albicans* strain ATCC 2091 were kindly provided by The Collection of Gause Institute of New Antibiotics (Moscow, Russia). The *Fusarium graminearum* strain VKM F-1668 was purchased from the All-Russian Collection of Microorganisms G.K. Skryabin Institute of Biochemistry and Physiology of Microorganisms, Russian Academy of Sciences (Pushchino, Moscow region, Russia).

### 2.2. Recombinant Peptide Production

#### 2.2.1. Expression Vector Construction

To produce a recombinant hybrid protein containing nigellothionin NsW2, a plasmideconjugated with *E. coli* thioredoxin via thepoly-histidine domain was constructed. The target PCR fragment was amplified using a forward primer containing a *KpnI* restriction site and a Met codon for CNBr cleavage and a reverse primer containing a *BamHI* restriction site and a stop codon. The PCR fragment was gel-purified, digested usingsuitable restriction enzymes, and cloned into the expression vector *pET-32b+* (Novagen, Madison, WI, USA), which was cut using the same restrictases to produce pET-32b-M-NsW2. The resulting construct was verified viaDNA sequencing.

#### 2.2.2. Fusion Protein Expression and Purification

*Escherichia coli* BL21 (DE3) cells transformed with the expression vector pET-32b-M-NsW2 were cultured at 37 °C in a Luria–Bertani medium containing 100 µg/mL of ampicillin untila culture density of D_600_ = 0.4–0.6 was reached. Expression was induced by adding isopropyl-b-D-thiogalactopyranoside (IPTG) to a concentration of 1 mM. The cells were cultured at room temperature (24 °C) overnight and harvested viacentrifugation (15 min at 4000× *g*). The pellets were suspended in the Lysis buffer for affinity chromatography (300 mM of NaCl, 50 mM of NaH_2_PO_4_ buffer, and 10 mM of imidazole; pH 8), and ultrasonicated for 1–1.5 h. The lysed cells were centrifuged (15 min at 14,000× *g*) to remove cell debris. The supernatant was applied to a preliminarily equilibrated column filled with Ni-NTA Sepharose (Thermo Fisher Scientific, Waltham, MA, USA). After the application of the cell lysate, the column was washed with three volumes of Wash buffer (300 mM of NaCl, 50 mM of NaH_2_PO_4_ buffer, and 20 of mM imidazole; pH 8.0) to remove non-specific components of the mixture. After that, the target protein trx-NsW2 was eluted with three volumes of the elution buffer (300 mM of NaCl, 50 mM of NaH_2_PO_4_ buffer, and 250 mM of imidazole; pH 8.0).

##### 2.3. Solid-Phase Extraction (SPE)

The Trx-NsW2 fusion protein was purified viasolid-phase extraction using a Manifold 10-valve instrument (Mecherey-Nagel, Düren, Germany). A C_18_ SPE cartridge was activated by buffer A (0.1% trifluoracetic acid (TFA) in MQ water). The eluate obtained after affinity chromatography was applied on the cartridge, and the target protein was eluted via anSPE elution buffer (60% acetonitrile in 0.1% TFA). The eluate was collected manually, and the column was washed with buffer B (80% MeCN in 0.1% TFA). The eluate was lyophilized, and the amount of trx-NsW2 was estimated gravimetrically.

### 2.4. Trx-NsW2 Purity Confirmation

#### 2.4.1. Analytical Reversed-Phase HPLC

The purity of the protein obtained viaSPE was confirmed viaRP-HPLC on a Jupiter C_5_ (300 Å; 5 µ; 250 × 4.6) analytical column (Phenomemex, Torrance, CA, USA) in a system consisting of a mobile phase of 0.1% TFA (solvent A) and 80% acetonitrile, 20% 2-propanole, and 0.1% TFA (solvent B). The analysis was carried in a linear gradient of buffer B. First of all, the column was equilibrated with 10% of solvent B. Then, its proportion was increased up to 50% in 15 min, to 75% in 10 min, and to 90% in 1 min. After that, the column was washed under 90% solvent B for 10 min at a flow rate of 1 mL/min, and its absorbance was monitored at 214 nm.

#### 2.4.2. SDS-PAAG Electrophoresis

The electrophoretic separation of the extract was carried out according to the protocol described. After affinity chromatography the eluate was subjected to SDS-PAGE which was applied to tris-glycine gels containing 15% acryl amide. The gels were stained with 0.4% Coomassie R250 Brilliant Blue (Thermo Fisher Scientific, USA) in 10% acetic acid and 40% methanol for 30 min, and the residual background stain was removed by immersing the gels in 10% acetic acid for 4 h.

#### 2.4.3. Edman Sequencing

N-terminal amino acid microsequencing was performed on an automated sequencer (PPSQ-33A model, Shimadzu Corporation, Kyoto, Japan) according to the manufacture’s protocol. Approximately 500 pmoles of the protein was taken for analysis. The identification of amino acid residues (PTH-derivatives) was performed using LabSolutions software (Shimadzu Corporation, Kyoto, Japan).

### 2.5. Protein Concentration Measurement

For a further measurement of the trx-NsW2 concentration in solutions, a Jupiter C_5_ (300Å 250 × 4.6) analytical column was calibrated. For this purpose, a series of two-fold protein dilutions were prepared in the range of 1000–62.5 µg/mL. The RP-HPLC process was carried under theabove-mentioned conditions. All measurements were obtained in triplicate. The results were processed using Agilent ChemStation software (Agilent Technologies, Santa Clara, CA, USA). The protein content was estimated via the integration of the area of the chromatographic peak corresponding to the protein.

### 2.6. Antifungal Assay

An antifungal assay was carried out according to the European Committee on Antimicrobial Susceptibility Testing (EUCAST) protocol [13]. Briefly, serial two-fold dilutions of the protein in double—strength RPMI 1640 (PanEco, Moscow, Russia) medium without the addition of Na_2_CO_3_with DMSO in the range of 10–0.312 µM were applied to 96-well microtiter plates (BioCell Technology, Irvine, CA, USA). Fungal spores (3 × 10^5^ CFU/mL) were applied to every well. Amphotericin B was applied as a positive control, and sterile water was used as a negative control. After 48 h of incubation, samples from each well were examined under a light microscope (Axio Scope A1 instrument (Carl Zeiss, Berlin, Germany) at 400-fold magnification. A disc-diffusion assay was applied as described previously [12]. Briefly, a fungal spore (3 × 10^5^ CFU/mL) suspension was spread on potato dextrose agar plates. Then, Trx-NsW2, at a concentration of 10 µM in 50% ethanol in water (*v*:*v*), was applied to 8 mm paper discs. Air-dried discs were placed on the seeded media. The antifungal effect was estimated after 24 and 48 h. All experiments were carried out in three independent repetitions with three replications.

### 2.7. Cytotoxic Assay

The MTT assay (a colorimetric test using 3-(4,5-dimethylthiazol-2-yl)-2,5-diphenyl-tetrazolium bromide) was performed on human lung adenocarcinoma A549, human pancreatic adenocarcinoma PANC-1, and human embryonic kidney Hek293 cell lines to measure the cytotoxicity of the trx-NsW2 peptide. First, 5.000 cells per well were seeded into a 96-well plate and incubated overnight in DMEM supplemented with 10% heat-inactivated fetal calf serum (FCS) and penicillin–streptomycin (10 IU·mL^−1^–100 μg·mL^−1^). The following day, fresh DMEM containing various concentrations of trx-NsW2 was added to the wells, and the plate was placed in an incubator under the following conditions: 95% humidity, 20% O_2_, 5% CO_2_, and 37 °C. After 48 h, 100 μL of DMEM and 20 μL of MTT reagent (0.5 mg·mL^−1^) were added to the wells and allowed to incubate for 1 h. The supernatant was then removed, the formazan crystals formed during MTT recovery by viable cells were dissolved in DMSO, and the optical density was measured using an Allsheng AMR-100 microplate photometer (China) at λ = 540 nm (subtracting the background optical density at λ = 700 nm) [14,15]. All experiments were conducted in two independent repetitions with five replications.

## 3. Results

### 3.1. Recombinant Protein Purification

**Figure 1:**
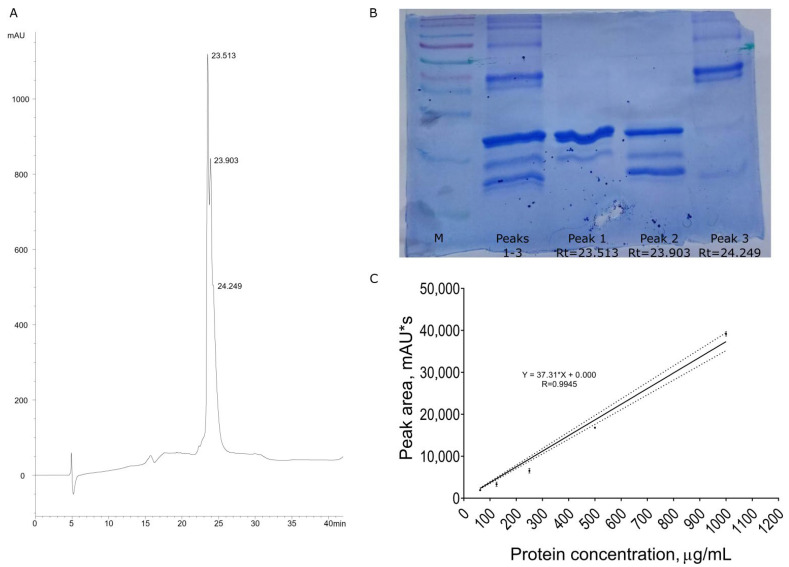
The production of the recombinant fusion trx-NsW2. (**A**) Analytical reversed-phase HPLC results for the desalted protein after Ni-NTA column (retention times for all peaks are indicated in minutes); (**B**) SDS PAGE results for the desalted protein after Ni-NTA column; (**C**) calibration graph for the recombinant fusion trx-NsW2.

The recombinant protein containing nigellothionin NsW2 and bacterial thioredoxin was purified from *E. coli* cells using a combination of affinity chromatography and solid-phase extraction. The protein’s purity was estimated viaRP-HPLC (Figure 1A). Interestingly, the target protein eluted as a triple peak, as shown in Figure 1B. To ascertain whether all distinguishable components belonged to trx-NsW2, all peaks were manually collected and subjected to SDS-PAAG electrophoresis. The results indicated that all peaks exhibited similar mobility, suggesting that they could be spatial isoforms of trx-NsW2. Notably, the third peak corresponded to a non-specific high molecular impurity, Figure 1B. To verify this observation, N-terminal automated microsequencing was performed on peaks 1 and 2.

It was determined that both peaks represented the same protein; however, the first peak exhibited a truncated N-terminal methionine residue which was partially cleaved by bacterial methionine aminopeptidase [16]. The relative amounts of the compound corresponding to peak 1 and peak 2 were 51.1 ± 3% and 38.1 ± 3%, respectively. Protein quantification was performed by integrating the peak squares using analytical RP-HPLC. A series of two-fold dilutions were analyzed, and the concentrations of trx-NsW2 were determined based on peak area integration. A calibration graph was generated, and a linear regression equation was calculated (Figure 1C). Based on these calculations, the total yield of the trx-NsW2 protein was determined to be 650 mg/L of bacterial culture.

### 3.2. Determination of Antifungal Activity

Firstly, the trx-NsW2 fusion protein was tested against three species of mycelial fungi (*A. niger*, *A. fumigatus*, and *F. graminearum*), and no inhibitory effects on conidial germination or hyphal growth wereobserved at an active concentration of 10 µM. However, the *C. albicans* culture was susceptible to the protein’ action, and the MIC value was recorded at 10 µM. Correspondingly, this indicates that this fusion protein lacks the strict antifungal properties observed in nigellothionin NsW2 itself [12,17], or that this effect may be limited due to the presence of the thioredoxin domain which leads to a decrease in the primary antifungal activity.

### 3.3. RegulatingFungal Growth and Revealing Cytotoxic Activity

**Figure 2:**
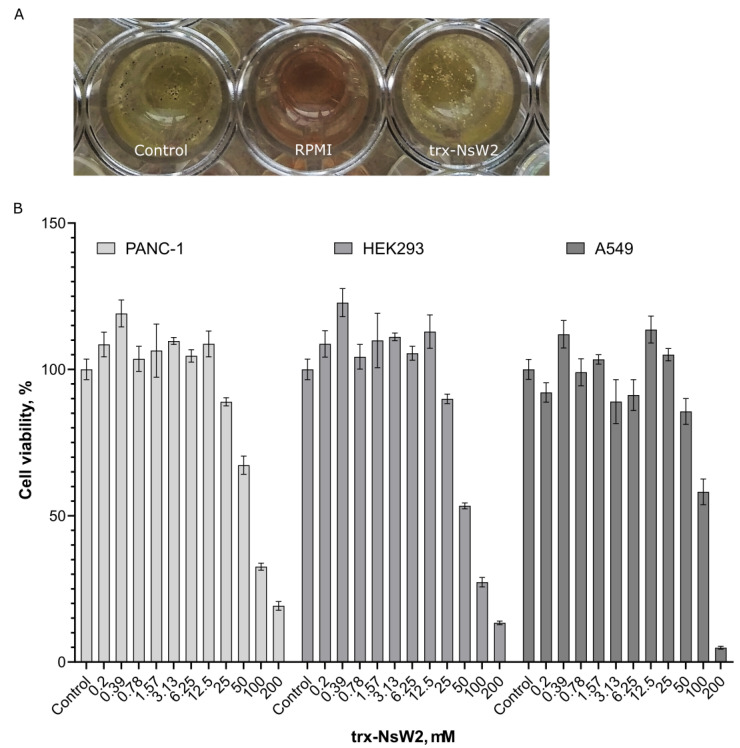
Biological activity reported for the recombinant fusion trx-NsW2. (**A**) Visualization of *A. niger* conidiogenesis after 48 h of incubation: MQ water (control), RMMI medium, and trx-NsW2 at 10 µM; (**B**) cytotoxic properties of trx-NsW2 *in vitro*.

At the next stage, *A. niger* was selected for further studies, and despite our previous results, a fungistatic effect was observed when the protein was presented at a concentration of 10 µM in the serial dilution assay. This effect manifested as a delay in spore germination during the initial 24 h of growth, but it was completely eliminated after 36 h of growth. Conidiogenesis was examined after 48–72 h of growth, revealing the formation of loose, pale conidiophores under the influence of 10 µM trx-NsW2, accompanied by the presence of white, fluffy mycelium (Figure 2A).

The cytotoxic activity of the trx-NsW2 fusion protein was evaluatedin vitro against three cell lines, two tumor cell lines and one non-tumor cell line, across a concentration range of 0.2–200 µM. Surprisingly, in all cases, there was an observed increase in cell proliferation, which manifested as a slight stimulation of culture growth (Figure 2B). This effect was observed over a wide range of active concentrations ranging from 0.2 to 25.0 µM. However, at higher concentrations of 100–200 µM, the trx-NsW2 exhibited significant cytotoxicity in all studied cell lines (Figure 2B).

## 4. Discussion

It is known that peptides from the thionin family of plant antimicrobial peptides (AMPs) possess strong antimicrobial activities due to cell membrane disruption. It is worth noting that this peptide has not demonstrated toxicity towards *E. coli* at concentrations below 26 µM [11]. This has made it possible to use a bacterial expression system. Previously, we showed that thenigellothionin NsW2 inhibits the growth of filamentous fungi and yeasts of both natural and clinical origins [12,17]. Moreover, thionins are known to have compact structures, making them thermostable and resistant to enzymatic cleavage. This makes thioninspromising molecules for use as analogs of antifungal antibiotics in technological processes [18]. Using the trx-NsW2 fusion protein as an antifungal agent could simplify the production process and make it more cost-effective. Here, we decided to investigate whether a fusion protein retains the antimicrobial properties of a single peptide. At the initial stage, a number of mycelial fungi and yeasts were tested for susceptibility to the trx-NsW2 action. This was carried out to compare activities between the single nigellothionin NsW2 and the chimeric protein. Previously, the NsW2 thionin was found to reveal MIC values at 0.77 µM against *Aspergillus* spp. [12] and 3.25 µM against *Candida albicans*, respectively [11]. In this study, trx-NsW2 was unable to demonstrate strong fungicidal activity compared to the native thionin. Then, *A. niger* was considered a convenient test subject as it is a well-studied fungus of significant importance to the industry (food spoilage, allergens, and the production of organic acids) [19]. According to existing data on the activity against *Ralstonia* of wheat nsLTPs as part of a fusion protein [20], it was expected that trx-NsW2 would also demonstrate antifungal properties. There is a single example of antifungal activity against filamentous phytopathogenic fungi using a hybrid recombinant protein containing thioredoxin linked with a fungistatic hevein-like peptide WAMP-3 with a chitin-binding domain against micelle forms of *Fusarium oxysporum* and *Bipolaris sorokiniana* [21].

Regarding the possible mode of fungistatic action, it should be noted that the black conidial pigment in *A. niger* conidia is represented by melanin, also known as aspergillin, which consists of two pigments: brown and green [22]. It can be assumed that trx-NsW2 or the peptide, as part of it, can inhibit one of the stages of the acetate–malonate melanin biosynthesis pathway [23]. The complete decolorization of the conidia suggests that the inhibition of melanogenesis occurred inthe initial stage. This could also be indicative of the disruption of gene expression, such as of the *wetA* gene, at the late stage of conidiophore formation [24]. It has been demonstrated that *wetA* mutants form normal conidiophores, but their conidia lack pigments [25].

Previously, we demonstrated that the native NsW2 peptide exhibited strong, dose-dependent cytotoxic activitiesagainst a range of tumor cell lines (AsPC-1, Colo357, RD, Jukart, HPF, B16, and HTC-116), with IC_50_values ranging from 0.2 to 0.4 µM [12,17]. Furthermore, the black seed thionin has been found to significantly suppress the transcriptional activity of certain oncogenes [26]. Therefore, it is of interest to compare trx-NsW2 with the previously obtained results. Interestingly, the fusion protein did not exhibit any inhibition towards the PANC-1, A549, and HEK293 cell lines at concentrations of up to 25–50 µM. This is the first report suggesting that the thioredoxin domain may play an important role in reducing the toxicity of membrane-active peptides. This effect is achieved via compensation for the high positive charge characteristic of NsW2, which is typical for thionins from class I, and results in alterations in membrane–peptide interactions [27,28].

## 5. Conclusions

Finally, the recombinant fusion protein described hereinis the first to demonstrate suppressive action against the food-spoiling fungus *Aspergillus niger* while exhibiting low levels of cytotoxicity toward mammalian cell cultures. This protein consists of a plant antimicrobial peptide from the thionin family linked with bacterial thioredoxin. The unique mechanism of action of this protein targets fungal physiology, which is uncommon for plant defense macromolecules in general. This represents the first report to demonstrate biological activity for a chimeric protein, making them potentially more attractive for biotechnology applications in food production and agriculture.

## Data Availability

The data in this study were available from the following sources the corresponding authors. These data are not publicly available due to the requirement to fund research projects.
